# Supplementary material for: Circulating levels of angiogenic factors and their association with preeclampsia among pregnant women at Mulago National Referral Hospital in Uganda
Source: PLoS One. 2021 May 19;16(5):e0251227. doi: 10.1371/journal.pone.0251227 (PMC8133410; doi:10.1371/journal.pone.0251227)
Supplement: S1 Datatool — (DOC) [file pone.0251227.s002.doc]

# CONTACT LOCATOR FORM

**STUDY TITLE: GENETIC AND ANGIOGENIC FACTOR PROFILES ASSOCIATED WITH PRE-ECLAMPSIA AMONG PREGNANT WOMEN IN MULAGO HOSPITAL, UGANDA**

**___________________________________________________________**

| Subject Study ID |  |
| --- | --- |

Hospital number ………………………………………………………………

Name ………………………………………………………………………….

Address (LC 1 Village, name of zone, division, sub county, district and whether is a tenant or not)

……………………………………………………………………….

……………………………………………………………………….

……………………………………………………………………….

……………………………………………………………………….

……………………………………………………………………….

Telephone numbers of mother, her spouse and another close friend or relative ……………………………………………………………

Date of birth ………………………………………………………………….

**IMPORTANT NOTE:** This page will be detached from the data collection form and kept separate in another file which will be kept under lock and key.

# QUESTIONNAIRE FOR RECRUITMENT

**STUDY TITLE: GENETIC AND ANGIOGENIC FACTOR PROFILES ASSOCIATED WITH PRE-ECLAMPSIA AMONG PREGNANT WOMEN IN MULAGO HOSPITAL, UGANDA**

**__________________________________________________________**

| Subject Study ID |  |
| --- | --- |

**SECTION A) DEMOGRAPHICS**

Qn 1

| Date of entry to study |  |  | _ |  |  | _ |  |  |  |  |
| --- | --- | --- | --- | --- | --- | --- | --- | --- | --- | --- |

**Day Month Year**

**Qn 2a**

| Date of Birth |  |  | _ |  |  | _ |  |  |  |  |
| --- | --- | --- | --- | --- | --- | --- | --- | --- | --- | --- |

**Day Month Year**

**Qn 2b.** Age (in completed years)

**Qn 3.** Religious affiliation (Circle what is applicable)

1. Catholic

2. Protestant

3. Muslim

4. SDA

5. Orthodox

6. Jehovah Witness

7. Born again

8. Others (specify) …………………………………………….

**Qn 4**. Marital status (Circle what is applicable)

1. Married

2. Single

3. Divorced or separated

4. Widowed

| Subject Study ID |  |
| --- | --- |

**SECTION B) ETHNICITY**

**Qn 5 a)** Tribe of baby’s parents

*Mother Father*

|  |  |
| --- | --- |

**5 b)** Tribe of baby’s grand-parents

*Maternal Paternal*

| G/mother | G/father | G/mother | G/father |
| --- | --- | --- | --- |
|  |  |  |  |

**SECTION C) SOCIAL HISTORY**

**Qn 6**. Smoking history [cigarettes or pipe] (Tick in the box for correct response)

1. Has never smoked

2. Stopped during index pregnancy

3. Smoked during index pregnancy

**Qn 7**. If reported smoking in the past or currently, frequency of smoking [cigarettes or pipe] (Tick in the box for correct response). **Smoke exposure is often measured in pack years (1 pack year = 1 year of smoking a pack of 20 cigarettes per day with conversion factors for other forms of tobacco.**

1. Smokes a pipe

2. If cigarette smoker, number of pack years

| Subject Study ID |  |
| --- | --- |

**Qn 8.** Consumption of alcohol in this pregnancy (Tick in the box for correct response)

1. No

2. Yes

**Qn 9**. If reports consumption of alcohol in this pregnancy, frequency of consumption

1. Bottles of beer per week

**OR**

2. Bottles of wine per week

**OR**

3. Bottles of spirits per week

**­SECTION D) MEDICAL HISTORY**

**Qn 10.** History of high blood pressure (hypertension)

No Yes Don’t know

**Qn 11**. History of heart failure

No Yes Don’t know

**Qn 12**. History of stroke

No Yes Don’t know

**Qn 13**. History of heart attack (myocardial infarction)

No Yes Don’t know

**Qn 14**. History of diabetes mellitus

No Yes Don’t know

| Subject Study ID |  |
| --- | --- |

**Qn 15.** HIV status Negative Positive

**Qn 16**. Family history of diabetes mellitus (type 1 or 2)

No Yes

**Qn 17**. Family history of pre-eclampsia

No Yes

**Qn 18**. Family history of hypertension

No Yes

**SECTION E) PAST OBSTETRIC HISTORY**

**Qn 19**. Is this your first pregnancy?

No Yes If Yes, skip to **Qn. 27**

**Qn 20**. Specify which order of pregnancy this is.

**Qn 21**. How many pregnancies have you carried beyond 7 months?

**Qn 22**. How miscarriages/ abortions or ectopic pregnancies have you had?

**Qn 23**. Have you delivered any baby of birth weight less than 2.5 Kg?

No Yes

| Subject Study ID |  |
| --- | --- |

**Qn 24**. Have you delivered any baby of birth weight more than 4.0 Kg?

No Yes

**Qn 25 a)** Have you been diagnosed with hypertension in a previous pregnancy?

No Yes

**25b)** If answer is **yes**, was this pre-eclampsia or not?

Yes No Don’t know

**Qn 26**. How long ago was your last pregnancy ended?

Years and Months

**SECTION F) GYNAECOLOGICAL HISTORY**

**Qn 27**. If this is your first pregnancy, what is the duration of the current relationship (in years)?

**Qn 28.** Is there history of condom use in this relationship?

No Yes

**Qn 29**. If answer to Qn 28 above is yes, how is the condom use classified? (tick in the appropriate box)

Consistently used (all the time) typically used (sometimes)

| Subject Study ID |  |
| --- | --- |

**Qn 30.** Is the partner different from one of the previous pregnancy?

No Yes NA

**Qn 31.** Do you have a history of infertility?

No Yes

**SECTION G) PRESENT PREGNANCY**

**Qn 32 a)** When was your last normal menstrual period?

| LNMP |  |  | _ |  |  | _ |  |  |  |  |
| --- | --- | --- | --- | --- | --- | --- | --- | --- | --- | --- |

**Day Month Year**

**Note:** If knows only month of LNMP state early (5th), Mid (15th) and late (25th).

**32 b)** Weeks of amenorrhea at the time of delivery.

-_

**Weeks days**

**Note:** If not sure of LNMP but has ultra sound results, use those to calculate the weeks of amenorrhoea.

**Qn 33.** Did you attend antenatal clinic in this pregnancy?

Yes No If No, skip to **Qn 35**

**Qn 34**. How many times did you attend the antenatal clinic?

| Subject Study ID |  |
| --- | --- |

**SECTION H) DELIVERY INFORMATION**

**Qn 35.** Date and time of delivery

| Date of delivery |  |  | _ |  |  | _ |  |  |  |  |
| --- | --- | --- | --- | --- | --- | --- | --- | --- | --- | --- |

**Day Month**  Year

| Time of delivery( 24 hour clock) |  |  | **:** |  |  |
| --- | --- | --- | --- | --- | --- |

**Hours Minutes**

**Qn 36.** Blood pressure (in mmHg) at admission for pre-eclamptics or at recruitment for controls

Systolic BP

Diastolic BP

**Qn 37.** Details of highest blood pressures recorded before delivery

(a)

| Date of highest systolic blood pressure |  |  | ­_ |  |  | _ |  |  |  |  |
| --- | --- | --- | --- | --- | --- | --- | --- | --- | --- | --- |

**Day Month Year**

(b)

| Date of highest diastolic blood pressure |  |  | ­_ |  |  | _ |  |  |  |  |
| --- | --- | --- | --- | --- | --- | --- | --- | --- | --- | --- |

**Day Month Year**

| Subject Study ID |  |
| --- | --- |

(c) Highest systolic blood pressure in mmHg

|  |
| --- |

(d) Highest diastolic blood pressure in mmHg

**Qn 38.** Dipstick urinary protein details

(a) Level of proteinuria at time of recruitment

Neg or trace 1+ 2+ 3+ 4+

(b) Highest level of proteinuria before delivery

Neg or trace 1+ 2+ 3+ 4+

(c)

| Date of highest urinary proteinuria (before delivery) |  |  | ­_ |  |  | _ |  |  |  |  |
| --- | --- | --- | --- | --- | --- | --- | --- | --- | --- | --- |

**Day Month Year**

**Qn 39.** Babies’ details

(1) Type of pregnancy Singleton Multiple

(a) If Singleton:

Baby's sex Male Female Ambiguous

Baby's weight (in Kgs) **.**

| Subject Study ID |  |
| --- | --- |

Foetal outcome: Live birth FSB MSB ENND

Comment if there are any obvious physical abnormalities on the baby

---------------------------------------------------------------------------------------------------------------------

(b) If Multiple pregnancy

|  | **Sex (M/F)** | **Baby’s weight (in kg)** | **Foetal outcome (Live birth, FBS, MSB, ENND)** | **Obvious physical abnormalities** |
| --- | --- | --- | --- | --- |
| 1^st^ baby |  |  |  |  |
| 2^nd^ baby |  |  |  |  |
| 3^rd^ baby |  |  |  |  |
| 4^th^ baby |  |  |  |  |
|  |  |  |  |  |

**Qn 40**. Mode of delivery

 Spontaneous vaginal  Spontaneous ventouse /forceps

 Induced vaginal  Induced ventouse /forceps

 Caesarean section

**Qn 41**. If caesarean section, indication/reason for it

-----------------------------------------------------------------------------------------------------------------------

**Qn 42.** Complications

 PPH  Eclampsia

 Abruption placentae  None

 Prematurity  Others (specify) …………………………

**Any other important comments**

------------------------------------------------------------------------------------------------------------------------------------------------------------------------------------------------------------------------------------------------------------------------------------------------------------------------------------

| Subject Study ID |  |
| --- | --- |

**I) MOTHER’S ANTENATAL BLOOD PRESSURE MEASUREMENTS**

| **Date of BP measurement** | **Gestational age** | **Blood pressure** | **Pulse rate** |
| --- | --- | --- | --- |
|  |  |  |  |
|  |  |  |  |
|  |  |  |  |
|  |  |  |  |
|  |  |  |  |
|  |  |  |  |
|  |  |  |  |
|  |  |  |  |
|  |  |  |  |
|  |  |  |  |
|  |  |  |  |

Name of interviewer: ---------------------------------------------------------

Signature of interviewer: ----------------------------------------------------

Date of interview: -------------------------------------------------------------
