# Supplementary material for: Circulating levels of angiogenic factors and their association with preeclampsia among pregnant women at Mulago National Referral Hospital in Uganda
Source: PLoS One. 2021 May 19;16(5):e0251227. doi: 10.1371/journal.pone.0251227 (PMC8133410; doi:10.1371/journal.pone.0251227)
Supplement: Attachment — Submitted filename: Response to Reviewers.pdf [file pone.0251227.s004.pdf]

Rebuttal letter

20th April 2021

Re: Resubmission of manuscript “Circulating levels of angiogenic factors and their association with preeclampsia among pregnant women at Mulago national referral hospital in Uganda”

Dr. Antonio Simone Laganà,
The academic Editor
PLOS ONE Journal

Dear academic editor:

We thank you for the opportunity to further revise our manuscript, **“Circulating levels of angiogenic factors and their association with preeclampsia among pregnant women at Mulago national referral hospital in Uganda”**. We appreciate the careful review and useful suggestions provided by the editor and reviewers. We believe that the manuscript is substantially improved after making the recommended edits. We deem it not necessary to submit laboratory protocols since we utilised commercially available kits and followed manufacturer’s guidelines to perform the assays. We also affirm that no retracted articles have been referenced in the manuscript.

Following this letter are the editor’s and reviewers’ comments with our responses. We have included a copy of the manuscript with track changes labelled “Revised Manuscript with Track Changes” and a revised manuscript without track changes labelled “Manuscript”.

Thank you for your consideration.

Sincerely,

Dr Annettee Nakimuli

On behalf of the authors

Editor's comments:

Editor: Journal Requirements: Please review your reference list to ensure that it is complete and correct. If you have cited papers that have been retracted, please include the rationale for doing so in the manuscript text, or remove these references and replace them with relevant current references. Any changes to the reference list should be mentioned in the rebuttal letter that accompanies your revised manuscript. If you need to cite a retracted article, indicate the article's retracted status in the References list and also include a citation and full reference for the retraction notice.

Response: We have reviewed the reference list and affirm that it is complete and correct. There are no retracted papers cited.

Reviewers' comments:

Reviewer #1:

Reviewer: THE SENTENCES NUMBERED FROM 205 TO 213 NEED CLARITY AS THEY ARE VITAL TO UNDERSTAND. PLEASE WRITE THE CORRECT INFORMATION AS THE SENTENCE FOR BOTH CASES AND CONTROLS ARE THE SAME.
Response: Thank you for this comment. We have corrected the sentences indicated and now read more clearly.

Reviewer: TOO MUCH TECHNICALITY ABOUT THE ASSAYS CAN EB CONSOLIDATED.
Response: We have summarised all the laboratory procedures under one paragraph "Plasma collection and immunoassays".

Reviewer: ELABORATION ABOUT TRANSLATION IN CLINICAL PRACTIE IS IMPORTANT TO MAKE THIS STUDY USEFUL.
Response: Thank you for this comment, we have addressed this in the discussion, lines 388 to 392 in the manuscript with track changes, to elaborate how the results obtained can be translated into clinical practice in Uganda.

Reviewer #3:

Reviewer: Table 5 - Why did the authors not include other variables in the model adjustment such as maternal age at pregnancy?
Response: Thank you so much for this comment. We started with a univariate analysis of all the variables against the dependent variable. Variables with a p-value less than 0.25 in univariate analysis were considered for the multivariate analysis. The variables used in the final model were obtained by backward elimination. We have added this to the main text, Lines 273 to 275 in the manuscript with track changes. Furthermore, maternal age could not be included in the model because it was one of the variables used to match cases to their controls.

Reviewer: Also add a footnote with the adjusted variables under the table 5.
Response: Thank you so much for this comment. Adjusted variables have been indicated under Table 5 as advised.
