# Supplementary material for: Circulating levels of angiogenic factors and their association with preeclampsia among pregnant women at Mulago National Referral Hospital in Uganda
Source: PLoS One. 2021 May 19;16(5):e0251227. doi: 10.1371/journal.pone.0251227 (PMC8133410; doi:10.1371/journal.pone.0251227)
Supplement: Attachment — Submitted filename: Response to Reviewers.docx [file pone.0251227.s003.docx]

**Rebuttal Letter**

**10^th^/February/2021**

**Re:** Resubmission of manuscript “Circulating levels of angiogenic factors and their association with preeclampsia among pregnant women at Mulago national referral hospital in Uganda”

**Dr. Antonio Simone Laganà,**

**The academic Editor**

**PLOS ONE Journal**

Dear academic editor:

We thank you for the opportunity to revise our manuscript, Circulating levels of angiogenic factors and their association with preeclampsia among pregnant women at Mulago national referral hospital in Uganda. We appreciate the careful review and useful suggestions provided by the editor and reviewers. We belief that the manuscript is substantially improved after making the recommended edits. Following this letter are the editor and reviewers’ comments with our responses. We have included a copy of the manuscript with track changes labelled “Revised Manuscript with Track Changes” and a revised manuscript without track changes labelled “Manuscript”.

Thank you for your consideration.

Sincerely,

Dr. Annettee Nakimuli

On behalf of the authors

**Editor Comments:**

**Editor:** Please ensure that your manuscript meets PLOS ONE's style requirements, including those for file Renaming. The PLOS ONE style templates can be found at

<https://journals.plos.org/plosone/s/file?id=wjVg/PLOSOne_formatting_sample_main_body.pdf> and

<https://journals.plos.org/plosone/s/file?id=ba62/PLOSOne_formatting_sample_title_authors_affiliations.pdf>

**Response:** We have reorganised all components of the manuscript to meet the PLOS One style requirements, including file renaming as guided by the templates at the provided links.

**Editor:** Please include additional information regarding the survey or questionnaire used in the study and ensure that you have provided sufficient details that others could replicate the analyses. For instance, if you developed a questionnaire as part of this study and it is not under a copyright more restrictive than CC-BY, please include a copy, in both the original language and English, as Supporting Information.

**Response:** We developed a data tool as part of the study. This was printed only in English and administered by a study nurse/midwife. We have included a copy of the data tool in the submission.

**Editor:** You indicated that you had ethical approval for your study. In your Methods section, please ensure you have also stated whether you obtained consent from parents or guardians of the minors included in the study or whether the research ethics committee or IRB specifically waived the need for their consent.

**Response:** As per the Uganda National Council for Science and Technology, pregnant teenage mothers above 14 years of age are considered emancipated minors capable of giving informed consent (https://www.uncst.go.ug/guidelines-and-forms/National Guidelines for Research Involving Humans as Research Participants). We therefore had permission from the IRB to include participants below 18 years in the study, however none of these were included because they were ineligible for various other reasons. We have made the required corrections in the methods section.

**Editor:** Please provide a sample size and power calculation in the Methods, or discuss the reasons for not performing one before study initiation.

**Response:** We have included a section “Sample size estimation” in the methods section to indicate the sample size and power calculations.

**Reviewers' comments:**

**Reviewer #1:**

**Reviewer:** The study has been conducted well to analyse the fact that these angio markers are raised in PE. The predictability is not assessed due to the fact that the inclusion criteria has been 20 weeks of gestation so the narration about this issue is out of context and needs to be modified.

**Response:** Thank you for this comment, we have revised/modified all the aspects of the manuscript to represent the correct context of this work, from the introduction up to the discussion.

**Reviewer:** Establishment of the fact that angiogenic markers are deranged in comparison to matched normal population is a good study. What you can do is look at the levels quantitatively and identify those one who have a count of 85 or more and compare them to find out whether they had a severe disease. this way rather than for prediction you can also understand its value in risk stratification which is clinically of great value. If you are interested you can request the gynecs in your institution to connect to me at [girijawagh@gmail.com](mailto:girijawagh@gmail.com) about a gestosis score validation may be can be done in-the same population through a different study with new ethics approval.

**Response:** Thank you for the suggestions for another study that can be carried out in this setting to investigate the role of angiogenic markers in risk stratification using a gestosis score. We are interested in pursuing this and we shall be getting in touch with you at the email provided for further discussion.

**Reviewer #2:**

**Reviewer:** This is a case control study that reflects the association of maternal circulating levels of VEGF, PlGF, sFlt1 and the sFlt1/PlGF ratio with PE, and compare their distribution both in normal healthy pregnancies as well as those with PE and to determine their predictive power of the disease among women in a Ugandan population. The uniqueness of the study is employing different methodological approaches such as Luminex assay, heat map, trained logistical regression model at 10 fold cross validation obtain high accurate predictive values of the angiogenic factors across different GA including validation. However the demographic as well as the clinical characteristics may needed further inclusion of variables which could give meaningful interpretation of the result such as Race, BMI, IVF, smoking history singleton or multiparity and mentioning exclusion number of total patients during analysis and thereby upgrading the tables -

**Response**: We have included family history of diabetes mellitus and smoking history to the clinical characteristics in Table 2. All participants self-reported to be Black Africans with both parents of African ancestry. Further ancestral delineation was not feasible. All participants had conceived naturally. We have adjusted the tables to include the total number of participants.

**Reviewer:** Moreover the definition of Preeclampsia needs to be stated clearly

**Response:** We have made corrections to the definition of Preeclampsia, stating; Preeclampsia was defined as new onset hypertension consisting of increased systolic BP of ≥ 140 mmHg and diastolic BP ≥ 90 mmHg on 2 different measurements at least 4 hours apart, plus proteinuria ≥ +1 on dipstick, at ≥ 20 weeks of gestation.

**Reviewer:** There are copple of sites where reference may needed such as lines 313,324,345

**Response:** We have made changes to reduce the discussion on VEGF as advised by another reviewer. Hence we deleted the above sentences requiring referencing. We have appropriately referenced the rest of the discussion.

**Reviewer:** Finally the manuscript needs to be written more concisely- for example methods section containing study population or study design/preparation could be merged and shortened in one paragraph; Also too much info on VEGF would not matters given that sFlt1/PlGF ratio itself has a good predictive value and gives similar result as other studies.

**Response:** We have made corrections and shortened the section on study design and study population.

**Reviewer#3:**

**General Comments**

**Reviewer:** The authors conducted a study assessing the predictive ability of angiogenic factors for pre-eclampsia. While this is an important area, this has been explored in several studies including in sub-Saharan countries (PMID: 28137987; PMID: 29523269); not referenced by the authors. Therefore, a clear justification of why this study was conducted and what it adds to the literature is needed, but not adequately provided.

**Response:** We have provided a better justification of the study and have also included the suggested references.

**Reviewer:** In general, the introduction is too long with a lot of basic information. Consider revising and focusing more on the focus of the study objective.

**Response:** Thank you for the comment, the introduction has been reduced and made more focused.

**Reviewer:** Line 87- 89: The authors state that BP and urine measurements are not specific to pre-eclampsia, therefore warranting assessment of angiogenic factors. I don’t agree with this statement as angiogenic factors are not specific to pre-eclampsia only. Please revise or justify reasoning more

**Response:** We have revised and also deleted that statement.

**Reviewer:** Table 1: Add the total number of cases and controls to the top rows

**Response**: We have included the total numbers of cases and controls.

**Reviewer**: The authors stated that they matched 106 each in cases and controls but some category numbers do not add up to this in the table e.g., gestational age.

**Response**: We have checked and updated Table 1 accordingly.

**Reviewer**: Why bother grouping age into 43-47 when only person falls under this category?

**Response**: We have changed age group 33-37 to Above 32 years. As such, the person previously grouped under 43-47 has been included in the Above 32 years category.

**Reviewer** The authors present adjusted ORs in Table 5 but do not state anywhere the variables that were adjusted.

**Response**: We have added a statement to reflect under the section; ‘Association of angiogenic factors with Preeclampsia’, “In the multivariate analysis, we adjusted for family history of preeclampsia and family history of hypertension”. This analysis has been included in Table 5.
